# Size-Dependent Immunomodulatory Effects of Fe_3_O_4_ Nanoparticles by Inducing Pro-Inflammatory Polarization of Macrophages to M1 Type

**DOI:** 10.3390/molecules31091492

**Published:** 2026-04-30

**Authors:** Yan Yang, Haoyu Yu, Mengying Fu, Hui Wang, Yang Yue, Lihua Geng, Quanbin Zhang, Jing Wang, Jiaqi Wan, Ning Wu

**Affiliations:** 1 College of Materials Science and Engineering, Qingdao University of Science and Technology, Qingdao 266042, China; 2023061027@mails.qust.edu.cn; 2 Chinese Academy of Sciences and Shandong (CAS) and Shandong Province Key Laboratory of Experimental Marine Biology, Institute of Oceanology, Chinese Academy of Sciences, Qingdao 266071, China; yuhy@qdio.ac.cn (H.Y.); fumengying23@mails.ucas.ac.cn (M.F.); wanghui225@qdio.ac.cn (H.W.); yueyang@qdio.ac.cn (Y.Y.); ihgeng@qdio.ac.cn (L.G.); qbzhang@qdio.ac.cn (Q.Z.); jingwang@qdio.ac.cn (J.W.); 3 Laboratory for Marine Drugs and Biological Products, Pilot National Laboratory for Marine Science and Technology (Qingdao), Qingdao 266237, China; 4 Nantong Zhongke Marine Science and Technology Research and Development Center, Nantong 226333, China

**Keywords:** Fe_3_O_4_ nanoparticles, macrophage polarization, size-dependent, tumor microenvironment

## Abstract

Tumor-associated macrophages (TAMs) are pivotal in shaping the immunosuppressive tumor microenvironment (TME). Reprogramming TAMs towards an anti-tumor M1 phenotype represents a promising strategy to enhance anti-tumor immunity. While Fe_3_O_4_ nanoparticles (NPs) possess immunomodulatory potential, the influence of NP size on macrophage polarization and the underlying mechanisms remain unclear. This study aims to systematically investigate the size-dependent immunomodulatory effects of Fe_3_O_4_ NPs and elucidate their mechanisms. We synthesized a series of Fe_3_O_4_ NPs of controlled sizes (5 nm, 10 nm, 30 nm, and 100 nm) via the polyol method. Among these, the 10 nm NPs demonstrated superior cellular uptake efficiency in macrophages. This enhanced uptake induced a significant increase in intracellular reactive oxygen species (ROS) levels. Subsequently, the elevated ROS activated the NF-κB signaling pathway, promoting M1 macrophage polarization. This polarization was evidenced by enhanced CD86 expression, increased nitric oxide (NO) release, and elevated secretion of pro-inflammatory cytokines. This study identifies 10 nm as the optimal size for Fe_3_O_4_ NPs to elicit their maximal immunomodulatory effects. Our findings establish a crucial size-design principle for the rational development of nano-immunotherapeutic agents and identify 10 nm Fe_3_O_4_ NPs as a promising candidate for TAM-targeted cancer therapy.

## 1. Introduction

Tumor progression and therapeutic resistance are governed not only by tumor cell heterogeneity but also by the profoundly immunosuppressive tumor microenvironment (TME). Within this complex ecosystem, tumor-associated macrophages (TAMs) frequently constitute the predominant immune population. TAMs typically exhibit an M2-like polarization state that actively promotes tumor growth, angiogenesis, immune evasion, and tissue remodeling [1]. This pro-tumorigenic polarization critically contributes to the failure of conventional therapies, including chemotherapy, radiotherapy, and even immune checkpoint inhibitors. Consequently, reprogramming TAMs from immunosuppressive M2 to immunostimulatory M1 phenotypes represents a highly promising strategy to reverse immunosuppression, enhance anti-tumor immunity, and improve therapeutic outcomes [2,3,4].

From a chemical perspective, nanoparticle-based platforms offer unprecedented opportunities for precise immune modulation due to their tunable physicochemical properties, including size, morphology, and surface chemistry [5]. Among these parameters, nanoparticle size exerts profound effects on cellular interactions [6,7,8], necessitating systematic investigations of size-dependent bioactivities. Iron oxide nanomaterials (e.g., Fe_3_O_4_) are particularly attractive for immunomodulation given their excellent biocompatibility, biodegradability, intrinsic magnetic properties, and unique capacity to participate in cellular iron metabolism and redox regulation via reactive oxygen species (ROS) [9,10]. Nevertheless, current research predominantly focuses on specific particle sizes: 15–30 nm for imaging applications [11], 45 nm for targeted drug delivery (PNE-PEG-TPP-Fe) [12], and >100 nm for TME regulation (SFT nanoparticles) [13]. A comprehensive understanding of how *fundamental* nanoparticle size governs macrophage interactions, downstream signaling, and immunophenotypic outcomes remains lacking. This knowledge gap impedes the rational “design-on-demand” of immunomodulatory nanomaterials.

Different preparation methods have significant effects on the size, morphology, crystallinity, magnetic properties, and surface chemical characteristics of nanoparticles [14] The co-precipitation method is the most traditional and simplest preparation method, producing Fe_3_O_4_ nanoparticles by mixing Fe^2+^ and Fe^3+^ salt solutions under alkaline conditions [15,16]. The chemical precipitation method can produce magnetite nanoparticles using only Fe^3+^ salt solutions under alkaline conditions [17]. This method is simple and low-cost, but the resulting nanoparticles have a wide size distribution and low crystallinity [14]. The thermal decomposition method involves decomposing metal precursors (such as iron acetylacetonate or iron carbonyl) in high-boiling-point organic solvents, which can produce monodisperse Fe_3_O_4_ nanoparticles with uniform size and high crystallinity, but the resulting nanoparticles are usually hydrophobic and require further surface modification [18,19]. The hydrothermal/solvent thermal method is conducted in a sealed high-pressure reactor, which imposes stringent requirements on equipment, poses challenges in reaction condition control, and has limited batch volume capacity, making it unsuitable for large-scale synthesis [20]. The polyol method features simpler operating conditions, as it does not require a sealed reactor for reactions under atmospheric pressure. It simultaneously utilizes polyols as both solvent and reducing agent to reduce iron precursors at relatively high temperatures, yielding iron oxide nanoparticles [21]. The advantages of the polyol method are the following: (1) the high boiling point of polyols allows the reaction to occur over a wide temperature range; (2) polyols can act as stabilizers, adsorbing on the nanoparticle surface to prevent aggregation; (3) the reaction conditions can be adjusted to accurately control the size and morphology of nanoparticles; and (4) the resulting nanoparticles have hydroxyl groups on their surface, which is beneficial for subsequent functional modifications.

Emerging evidence indicates Fe_3_O_4_ nanoparticles can polarize macrophages toward anti-tumor M1 phenotypes within the TME, thereby improving immunosuppression and exerting synergistic anti-tumor effects [22,23]. However, the mechanisms driving this polarization remain incompletely resolved. As professional phagocytes, macrophages internalize Fe_3_O_4_ nanoparticles, triggering intrinsic physicochemical property-driven stress responses essential for M1 polarization. The current understanding of the chemical biology underlying this process include the following:

(1) Direct iron signaling: Lysosomal degradation releases Fe^2+^/Fe^3+^ ions that activate pathways such as IRF5 [12,24].
(2) ROS-mediated signaling: Intracellular Fe^2+^ fuels Fenton reactions, generating ROS that promote polarization [25,26].

Based on these considerations, this study aims to: Systematically investigate the influence of Fe_3_O_4_ nanoparticle size on macrophage reprogramming efficacy and elucidate underlying mechanisms. We hypothesize that an optimal size window exists for Fe_3_O_4_ nanoparticles to maximize accumulation, iron release kinetics, and ROS generation in macrophages, thereby co-activating pro-inflammatory signaling. To test this, we synthesized size-controlled Fe_3_O_4_ nanoparticles (5, 10, 30, 100 nm) via the polyol method and stabilized them with sodium tripolyphosphate (STPP) coating. In this work, we not only identified the optimal size for promoting M1 polarization but also elucidated the underlying “uptake-ROS-signaling pathway-immunity/death” cascade for Fe_3_O_4_ nanoparticles. By establishing a clear structure-activity relationship based on nanoparticle size, this work provides critical chemical design principles for next-generation nano-immunomodulators and advancing Fe_3_O_4_ nanoparticle applications in cancer therapy.

## 2. Results

### 2.1. Preparation and Characterization of Fe_3_O_4_ Nanoparticles

**Figure 1:**
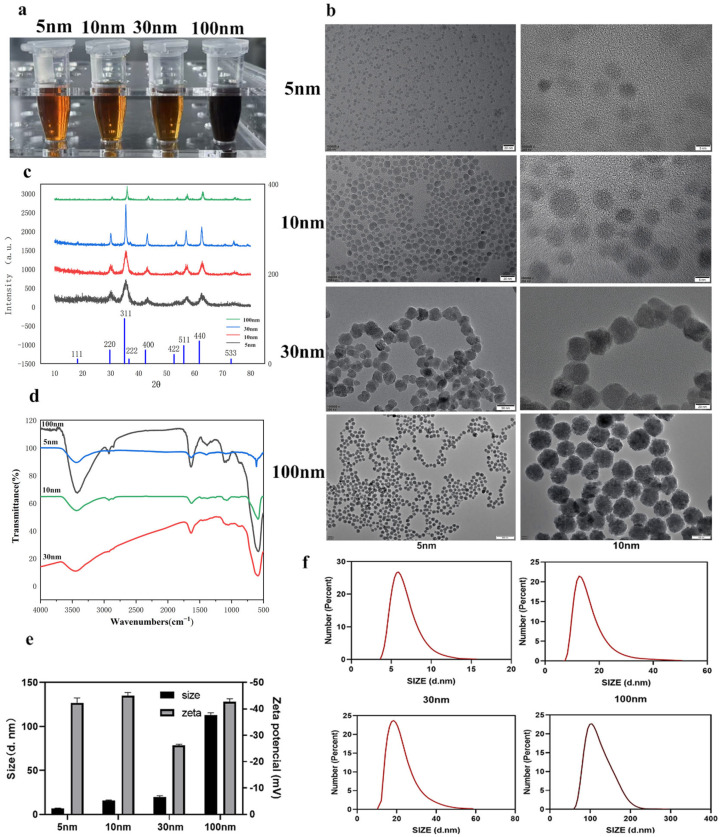
Physicochemical characterization of STPP-coated Fe_3_O_4_ nanoparticles with different sizes. (**a**) Photographs of Fe_3_O_4_ nanoparticle solutions. (**b**) Representative TEM images of Fe_3_O_4_ nanoparticles. (**c**) XRD patterns of the synthesized nanoparticles. (**d**) FT-IR spectra of Fe_3_O_4_ nanoparticles. (**e**,**f**) Hydrodynamic size distribution and zeta potential analysis of STPP-coated Fe_3_O_4_ nanoparticles. Data are presented as mean ± S.D. Statistical significance was determined by one-way ANOVA followed by Tukey’s post hoc test.

Four Fe_3_O_4_ nanoparticles with different sizes (5 nm, 10 nm, 30 nm, and 100 nm) were successfully prepared via the polyol method, and their surfaces were successfully coated with sodium tripolyphosphate (STPP) through electrostatic adsorption (Figure 1a). Compared with citrate coating, sodium tripolyphosphate (STPP) coating can provide a denser, more stable negatively charged surface, and exhibits better colloidal stability under physiological conditions [27]. Literature indicates that STPP coating can significantly enhance cellular uptake efficiency and promote cytoplasmic distribution, avoiding lysosomal retention [28], whereas citrate coating is prone to desorption, aggregation, and may inhibit inflammatory factors [29]. Therefore, using STPP to achieve negative surface charge is a more reasonable choice. All nanoparticles except the 100 nm samples formed clear, homogeneous brownish suspensions; in contrast, the 100 nm formulation exhibited a darker, opaque appearance, suggesting slightly lower colloidal stability in aqueous media.

Transmission electron microscopy (TEM) images (Figure 1b) revealed spherical morphologies with narrow size distributions and no visible aggregation, confirming successful size control. X-ray diffraction (XRD) patterns (Figure 1c) matched the standard magnetite (Fe_3_O_4_) reference [30], with distinct diffraction peaks corresponding to the (220), (311), (400), (422), (511), and (440) crystal planes, confirming the cubic spinel structure [31].

FT-IR spectroscopy (Figure 1d) identified characteristic vibrational modes: C–H and C–O stretching bands at 2900–2850 cm^−1^ and 1100 cm^−1^, respectively, indicating the presence of residual polyol ligands. A strong Fe–O vibrational band at ∼585 cm^−1^ confirmed the formation of the magnetite phase [30,32]. After STPP coating, the zeta potential of all nanoparticles shifted to a stable negative range (−25 to −45 mV; Figure 1e), confirming successful electrostatic surface modification.

Dynamic light scattering (DLS) measurements yielded hydrodynamic diameters of 6.09 ± 1.52 nm (ultrasmall-5 nm), 16.42 ± 5.12 nm (10 nm), 24.74 ± 8.99 nm (30 nm), and 113.5 ± 30.41 nm (100 nm) (Figure 1f). All formulations remained stable in deionized water for over three months without aggregation, demonstrating excellent colloidal stability.

Collectively, these results confirm that Fe_3_O_4_ nanoparticles with varying sizes, when uniformly coated with sodium tripolyphosphate (STPP), exhibit consistent crystallinity, surface chemistry, and stability, enabling reliable comparison of size-dependent biological effects.

### 2.2. Cytotoxicity of Fe_3_O_4_ Nanoparticles

**Figure 2:**
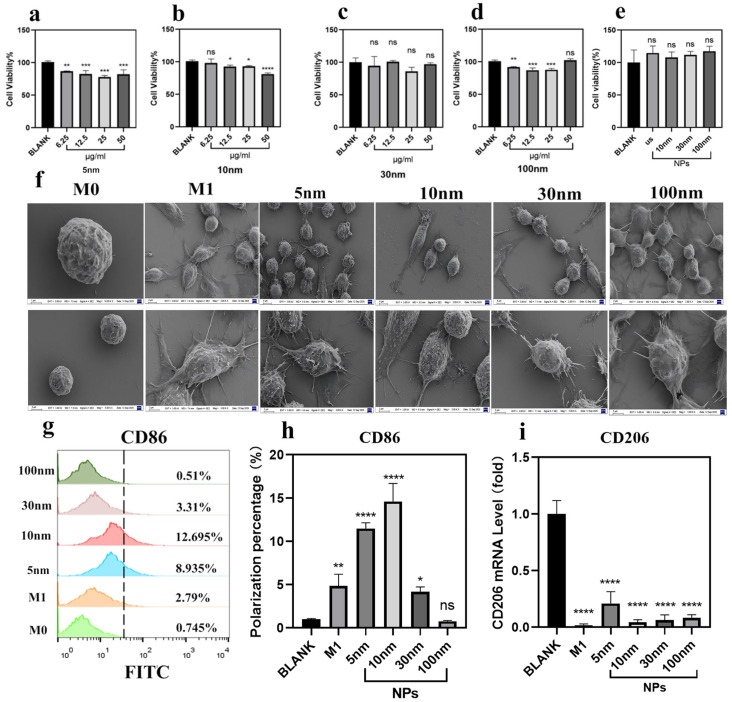
Effects of different-sized Fe_3_O_4_ nanoparticles on M1 polarization of RAW264.7 macrophages. (**a**–**d**) Viability of HaCaT cells treated with various concentrations of (**a**) 5 nm, (**b**) 10 nm, (**c**) 30 nm, and (**d**) 100 nm Fe_3_O_4_ nanoparticles for 24 h, as determined by the MTT assay. (**e**) Viability of RAW264.7 macrophages after nanoparticle treatment. (**f**) Representative SEM images showing the morphological changes in RAW264.7 cells after 24 h treatment with nanoparticles. (**g**,**h**) Flow cytometry analysis of CD86-positive cell populations in RAW264.7 cells treated with nanoparticles for 24 h. (The dashed line in the figure serves as a reference point where the CD86-positive rate on the right side of M0 approaches zero). (**i**) RT-qPCR analysis of CD206 gene expression levels. Data are presented as mean ± S.D. Statistical significance was determined by one-way ANOVA followed by Tukey’s post hoc test. **** *p* < 0.0001, *** *p* < 0.001, ** *p* < 0.01, * *p* < 0.05, ns = not significant was considered statistically significant.

The biocompatibility of STPP-coated Fe_3_O_4_ nanoparticles was evaluated using the MTT assay in HaCaT keratinocytes. Cells were exposed to nanoparticle concentrations ranging from 6.25 to 50 µg/mL for 24 h. As shown in Figure 2a–d, all nanoparticle sizes induced minimal cytotoxicity. Even at the highest concentration (50 µg/mL), cell viability remained above 80% for all groups, with no statistically significant differences compared to untreated controls (*p* > 0.05).

Similarly, in RAW264.7 macrophages, viability was unaffected by nanoparticle exposure at 6.25 µg/mL (Figure 2e), with a slight (non-significant) increase in viability observed across all sizes (*p* > 0.05). These findings indicate that the STPP-coated Fe_3_O_4_ nanoparticles are non-toxic to both non-immune and immune cells under the tested conditions, supporting their suitability for subsequent immunomodulatory studies.

### 2.3. Fe_3_O_4_ Nanoparticles Polarizes Macrophages to CD86^+^ M1 Phenotypz

To evaluate the impact of nanoparticle size on macrophage polarization, we first assessed morphological changes via scanning electron microscopy (SEM). Undifferentiated RAW264.7 cells (M0 phenotype) exhibited a rounded, smooth surface (Figure 2f, control). In contrast, LPS/IFN-γ-stimulated M1 macrophages displayed a flattened, elongated morphology with prominent pseudopodia, indicative of an activated state. Similarly, all Fe_3_O_4_-stimulated macrophages adopted an elongated, spindle-like shape with extensive surface protrusions—a hallmark of M1 polarization (Figure 2f).

Next, we quantified M1 polarization using the surface marker CD86 by flow cytometry. As shown in Figure 2g,h, all nanoparticle sizes increased the proportion of CD86^+^ macrophages compared with the untreated control group. Notably, the response induced by 10 nm Fe_3_O_4_ nanoparticles was the strongest: CD86^+^ cells increased from 0.745% (control) to 12.695% (*p* < 0.001), significantly higher than the 5 nm (8.935%, *p* < 0.001), 30 nm (5.12%, *p* < 0.05), and 100 nm (2.31%, *p* > 0.05) samples.

Conversely, expression of the M2 marker CD206 was significantly downregulated in all nanoparticle-treated groups compared to untreated controls, with the greatest reduction observed in the 10 nm group (Figure 2i). This bidirectional modulation—upregulation of M1 markers and suppression of M2 markers—confirms that Fe_3_O_4_ nanoparticles induce a robust, size-dependent shift toward classical M1 polarization, with 10 nm particles exhibiting the highest efficacy.

### 2.4. Cellular Uptake of Different Sized Nanoparticles

**Figure 3:**
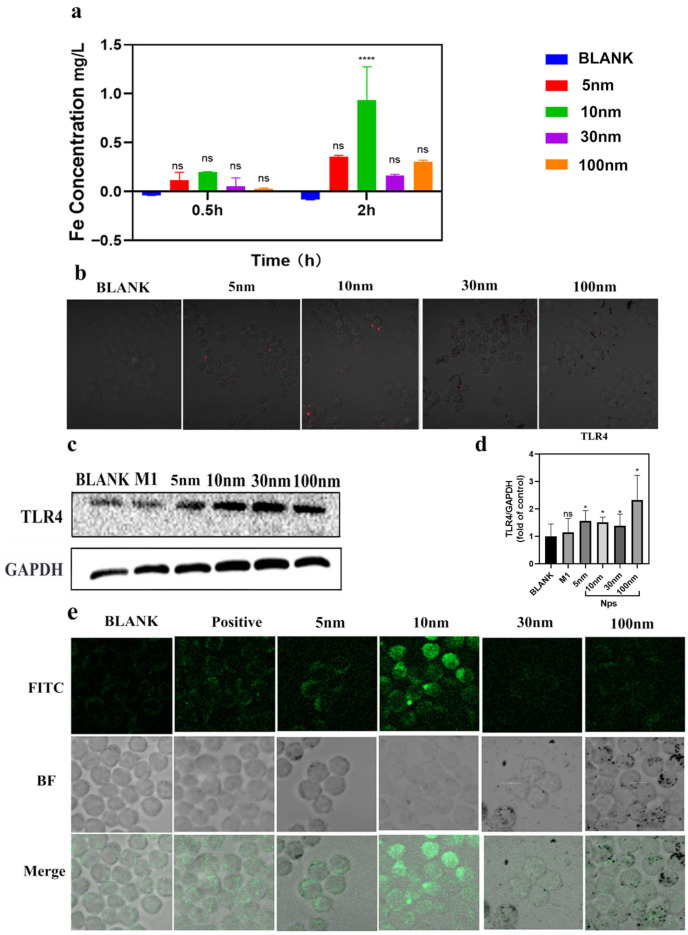
Uptake of Fe_3_O_4_ nanoparticles with different particle sizes by macrophages and their effect on intracellular reactive oxygen species generation. (**a**) Iron content in RAW264.7 cells at 0.5 h and 2 h, as measured by Inductively Coupled Plasma (ICP) test. (**b**) Fluorescence image of Fe_3_O_4_ magnetic nanoparticles in RAW264.7 macrophages at 2 h. Red fluorescence indicates iron ions. (**c**) Western blot analysis of TLR4 expression in RAW264.7 cells co-cultured with Fe_3_O_4_ magnetic nanoparticles of different particle sizes for 24 h (*n* = 3). (**d**) The expression levels of TLR4 in RAW264.7 cells. (**e**) Detection of reactive oxygen species levels in macrophages after 24 h of treatment with magnetic nanoparticles of different sizes. Green fluorescence indicates DCFH-DA staining. Data are expressed as mean ± S.D. for each data point. Statistically significant differences are indicated as **** *p* < 0.0001, * *p* < 0.05, ns = not significant.

To elucidate the mechanism underlying the superior immunomodulatory activity of 10 nm nanoparticles, we quantified intracellular iron accumulation using inductively coupled plasma optical emission spectrometry (ICP-OES). RAW264.7 cells were incubated with nanoparticles for 0.5 h and 2 h. At both time points, iron content increased in a time-dependent manner. The blank group’s content is very low, below the instrument’s detection limit, showing as not detected. The appearance of negative values is due to the very low concentration of the element being tested in the sample, generated by the instrument’s own noise, which is a normal phenomenon. Crucially, at 2 h, cells treated with 10 nm Fe_3_O_4_ NPs accumulated 3.1-fold more intracellular iron than 5 nm NPs, 4.9-fold more than 30 nm NPs and 3.4-fold more than 100 nm NPs (*p* < 0.01) (Figure 3a), indicating optimal uptake efficiency at the 10 nm scale.

Fluorescence imaging using Cy5.5-labeled Fe_3_O_4_-STPP nanoparticles further confirmed this trend. After 2 h incubation, the strongest red fluorescence signal was observed in cells treated with 10 nm NPs, corroborating the ICP-OES results (Figure 3b).

Since TLR4 is involved in the recognition and internalization of nanoparticles [33,34], we detected the expression level of TLR4 protein using Western blot. As shown in Figure 3c,d, compared with the control group, all Fe_3_O_4_ nanoparticles upregulated TLR4 expression, suggesting that the recognition and uptake of nanoparticles may be enhanced through TLR4-mediated endocytosis.

Given the role of intracellular iron in Fenton-mediated ROS generation [35,36], we measured ROS levels using the fluorescent probe DCFH-DA. Confocal microscopy revealed that only treatment with 10 nm Fe_3_O_4_ NPs triggered a dramatic increase in green fluorescence intensity after 24 h (*p* < 0.001), far exceeding levels in other size groups and even the positive control (ROSUP, Figure 3e). This robust ROS burst was directly correlated with the highest intracellular iron uptake.

Combining these findings we found the optimal uptake at 10 nm and highest iron accumulation in cells which made maximal ROS generation. And we conclude that ROS serves as a central signaling molecule driving M1 polarization in response to 10 nm Fe_3_O_4_ nanoparticles. This establishes a clear mechanistic cascade: Size-dependent uptake → Iron overload → Fenton-driven ROS burst → TLR4/NF-κB activation → M1 polarization.

### 2.5. Fe_3_O_4_ Nanoparticles Induce Pro-Inflammatory Cytokine Secretion and Activate the TLR4/MyD88/NF-κB Signaling Axis

M1 and M2 macrophages secrete distinct cytokine profiles that critically determine their pro- or anti-tumor functions. To evaluate the immunomodulatory potency of Fe_3_O_4_ nanoparticles, we quantified the secretion of nitric oxide (NO), interleukin-6 (IL-6), and tumor necrosis factor-alpha (TNF-α) in RAW264.7 cell supernatants following 24 h of exposure to nanoparticles of varying sizes.

**Figure 4:**
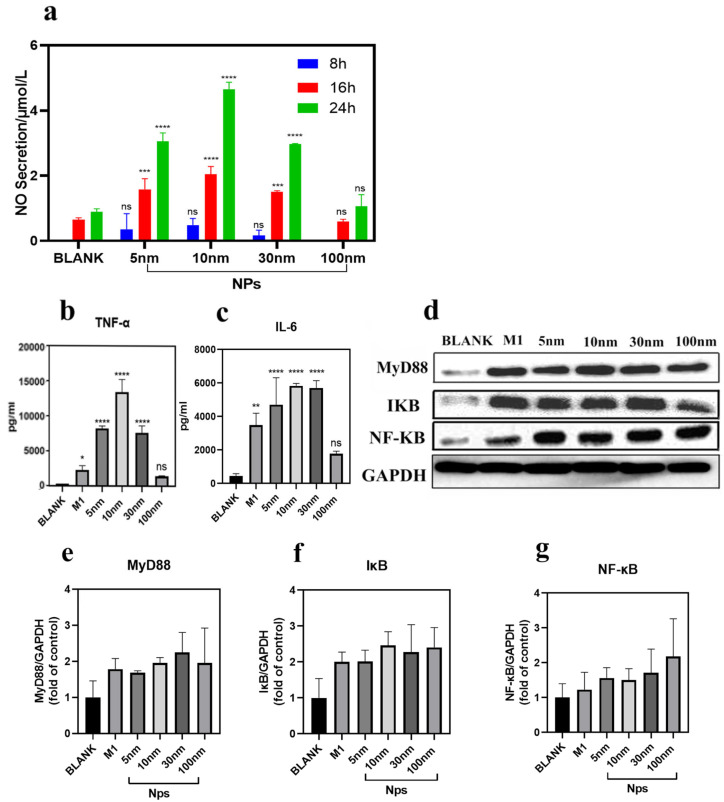
Fe_3_O_4_ magnetic nanoparticles promote macrophage polarization by activating the NF-κB pathway. (**a**) Nitric oxide (NO) production in RAW264.7 cells treated with nanoparticles at different time points. (**b**,**c**) Measurement of inflammatory mediators TNF-α and IL-6 in cell supernatants after treating RAW264.7 cells with magnetic nanoparticles of different particle sizes for 24 h. (**d**) Co-culture of RAW264.7 cells with Fe_3_O_4_ magnetic nanoparticles of different particle sizes for 24 h, *n* = 3. Western blot analysis of NF-κB, MyD88, and IκBα expression. Expression levels of (**e**) MyD88, (**f**) IκB, and (**g**) NF-κB in RAW264.7 cells. Data are expressed as mean ± S.D. for each data point. Statistically significant differences are indicated as **** *p* < 0.0001, *** *p* < 0.001, ** *p* < 0.01, * *p* < 0.05, ns = not significant.

As shown in Figure 4a, all nanoparticle sizes induced a time-dependent increase in NO production compared to untreated controls (Blank), with the peak response observed at 24 h. The 10 nm Fe_3_O_4_ nanoparticles elicited the strongest NO release significantly exceeding levels in the, 5 nm 30 nm, and 100 nm groups at all time points (8, 16, and 24 h; *p* < 0.01 for 10 nm vs. others). The 100 nm nanoparticles showed only marginal NO elevation, comparable to untreated cells (*p* > 0.05). 5 nm and 30 nm NPs induced intermediate levels of NO secretion.

Similarly, 24 h ELISA analysis demonstrated significant upregulation of TNF-α and IL-6 in all nanoparticle-treated groups compared to the blank control (*p* < 0.001; Figure 4b,c). The 10-nanometer Fe_3_O_4_ nanoparticles induced the highest cytokine secretion levels. Notably, these levels were comparable to or even higher than those induced by the positive control LPS/IFN-γ (M1 polarization stimulation), indicating significant M1 polarization capacity (*p* < 0.01 for IL-6 group versus LPS/IFN-γ group).

To elucidate the underlying signaling mechanism, we assessed activation of the TLR4/MyD88/NF-κB pathway via Western blotting. As depicted in Figure 3c and Figure 4d–g, treatment with all Fe_3_O_4_ nanoparticles induced upregulation of key signaling proteins: TLR4, MyD88, IκBα, and NF-κB p65.

These results indicate significant activation of the TLR4/MyD88/NF-κB inflammatory axis in macrophages treated with ferrous oxide magnetic nanoparticles of varying particle sizes, which is consistent with the phenomenon that ferrous oxide magnetic nanoparticles within the size range of 5 nm to 100 nm can drive M1 phenotype polarization.

### 2.6. Fe_3_O_4_ Nanoparticles Indirectly Suppress Tumor Cell Viability via M1 Macrophage-Mediated Paracrine Signaling

To validate whether Fe_3_O_4_ nanoparticles-induced M1 macrophages exhibit antitumor effects, we pretreated RAW264.7 macrophages with nanoparticles for 24 h. The supernatant was then collected as conditioned medium (CM) at concentrations of 6.25 µg/mL, 12.5 µg/mL, 25 µg/mL, and 50 µg/mL for 24 h. Subsequently, conditioned media treated with different concentrations and particle sizes were used to treat HCT116 and HT29 cells, and changes in the viability of both cancer cell lines were assessed.

**Figure 5:**
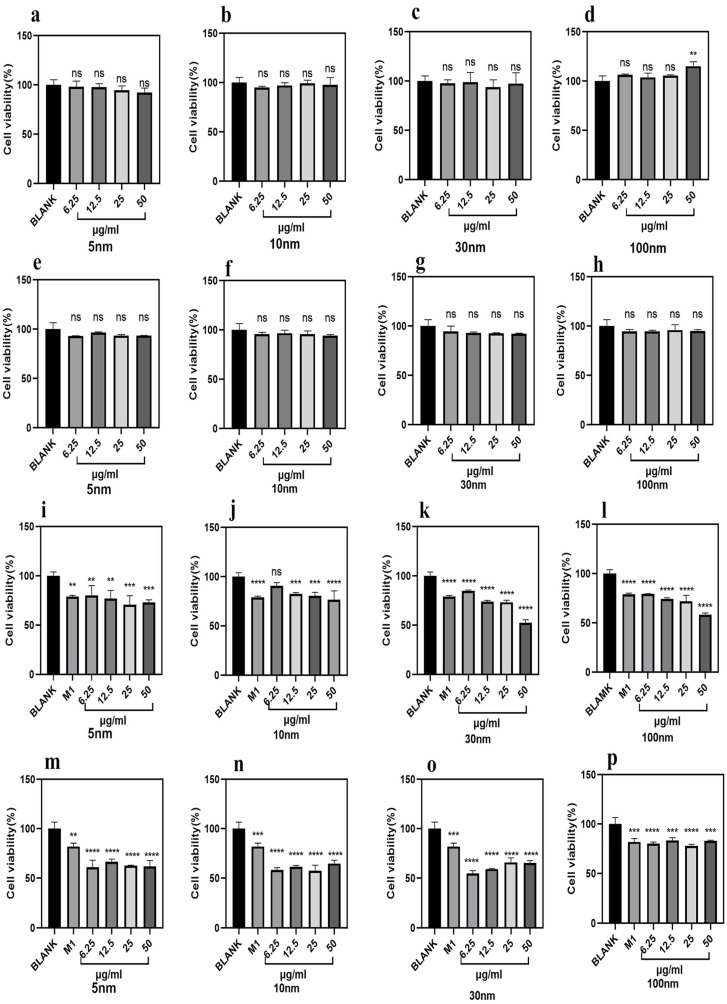
Different-sized Fe_3_O_4_ magnetic nanoparticles suppress the viability of HCT116 and HT29 cells by polarizing macrophages toward the M1 phenotype. Cytotoxicity of HCT116 cells treated with unmodified (us) (**a**), 10 nm (**b**), 30 nm (**c**), and 100 nm (**d**) Fe_3_O_4_ nanoparticles; HT29 cells treated with unmodified (us) (**e**), 10 nm (**f**), 30 nm (**g**), and 100 nm (**h**) Fe_3_O_4_ nanoparticles; HCT116 cells treated with unmodified (us) (**i**), 10 nm (**j**), 30 nm (**k**), and 100 nm (**l**) CMS; and HT29 cells treated with unmodified (us) (**m**), 10 nm (**n**), 30 nm (**o**), and 100 nm (**p**) CMS for 24 h. Data are mean ± SD, *n* = 3; **** *p* < 0.0001, *** *p* < 0.001, ** *p* < 0.01, ns = not significant vs. Blank or untreated tumor cells.

First, we confirmed that Fe_3_O_4_ nanoparticles themselves exhibited no direct cytotoxicity toward HCT116 or HT-29 cells after 24 h of exposure (Figure 5a–h). Cell viability remained >90% at concentrations up to 50 µg/mL, confirming that observed anti-tumor effects are indirectly mediated by reprogrammed macrophages.

When tumor cells were exposed to conditioned medium from M1-polarized macrophages, inhibition of cell viability was observed. As shown in Figure 5i–p:

HCT116 cells treated with conditioned medium containing magnetic nanoparticles of different particle sizes showed a significant reduction in cell viability. Specifically, the cell survival rate decreased with the increasing concentration of Fe_3_O_4_ magnetic nanoparticles: the survival rate of cells treated with 5 nm Fe_3_O_4_ magnetic nanoparticles decreased from the initial 100% to 70%, which was consistent with that of cells treated with 10 nm Fe_3_O_4_ magnetic nanoparticles (also decreased to 70%). In contrast, the survival rate of HCT116 cells treated with 30 nm Fe_3_O_4_ magnetic nanoparticles decreased to 55%, while that of cells treated with 100 nm Fe_3_O_4_ magnetic nanoparticles decreased to 60%.

Similarly, the viability of HT29 cells also exhibited a consistent downward trend after treatment with Fe_3_O_4_ magnetic nanoparticles, and the reduction extent was more significant compared with HCT116 cells. However, no obvious concentration-dependent trend was observed in HT29 cells. With the increase in Fe_3_O_4_ magnetic nanoparticle concentration, the viability of HT29 cells treated with 5 nm particles generally decreased to the range of 55–67%, while that of HT29 cells treated with 10 nm particles decreased to 50–65%. After treatment with 30 nm Fe_3_O_4_ magnetic nanoparticles, the survival rate of HT29 cells decreased to 55–70%. In addition, the survival rate of HT29 cells exposed to 100 nm Fe_3_O_4_ magnetic nanoparticles was reduced to 77–85%.

Analysis of the comprehensive experimental results revealed that nanomaterials with particle sizes ranging from 5 nm to 100 nm exhibited significant inhibitory effects on the growth of colon cancer cells. Notably, even at concentrations as low as 12.5 μg/mL, their inhibitory efficacy matched or surpassed that of positive controls, with inhibition rates reaching 30% for HCT116 cells and 40–50% for HT29 cells. These findings demonstrate that the nanomaterials can achieve high anti-colon cancer activity at relatively low doses.

## 3. Discussion

The phagocytosis of nanoparticles by cells is a complex biological process regulated by multiple factors, and there are significant differences in the ability and manner in which different types of cells phagocytose nanoparticles. Professional phagocytic cells have a significantly stronger ability to engulf nanoparticles compared to non-phagocytic cells. Sudduth et al. (2024) found that both macrophages and dendritic cells can efficiently uptake nanoparticles, but they have different preferences for surface charge [37]. Liao et al. (2025) further revealed that clathrin-mediated endocytosis requires particle clusters with a volume equivalent to approximately 90 nm particles, explaining why small particles can achieve efficient uptake through a synergistic effect [38]. Additionally, Claudia et al. (2017) directly compared and showed that macrophages uptake 200 nm polystyrene particles far more than alveolar epithelial cells [39]. In summary, cell type and particle size together determine the endocytosis efficiency of nanoparticles, and the optimal particle size for uptake varies among different cells.

This study, through rigorous comparison across a size series, clearly reveals a strong size-dependency in the immunomodulatory function of Fe_3_O_4_ nanoparticles and identifies ~10 nm as the optimal size for inducing M1 macrophage polarization.

This finding is substantiated by multi-dimensional data including cellular uptake, ROS generation, cytokine secretion, and surface marker expression. More importantly, it moves beyond phenomenological observation by establishing a quantitative link between a fundamental physical property of nanomaterials (size) and a complex biological function (immunophenotype regulation).

Our preliminary research has demonstrated that under the influence of external magnetic fields [40], magnetic nanoparticles containing iron oxide (Fe_3_O_4_) can achieve tumor-targeted accumulation through magnetic targeting properties. This mechanism enhances macrophage uptake of nanoparticles within the tumor microenvironment, thereby promoting macrophage polarization toward the M1 phenotype. These findings indicate that iron oxide magnetic nanoparticles maintain high uptake efficiency even in vivo, demonstrating significant clinical translation potential.

Our mechanistic investigation elucidates a clear cascade. First, the ~10 nm size likely represents an optimal scale for clathrin-mediated endocytosis in macrophages, leading to the highest internalization efficiency and iron load. Subsequent lysosomal degradation releases iron ions, which efficiently catalyze ROS production via the Fenton reaction. This intense oxidative stress acts as a critical second messenger, triggering sustained activation of the TLR4/MyD88-dependent NF-κB signaling pathway [41]. Activated NF-κB then drives the transcription and secretion of key pro-inflammatory cytokines like IL-6 and TNF-α, ultimately locking macrophages into the M1 phenotype [42]. This complete chain from “physical uptake” to “chemical signaling” to “gene expression and phenotypic fixation” profoundly illustrates the complex process by which nanomaterials regulate cell fate through metabolism-signal coupling.

Hedl et al. (2019) found that in human M1 macrophages, IRF5 promotes ROS production by regulating the expression of NADPH oxidase subunits such as p40phox, p47phox, and p67phox, and this process depends on IRF5’s activation of the MAPK, NF-κB, and Akt2 pathways [43]. On the other hand, IKKβ can phosphorylate and activate both IRF5 (at Ser462) and NF-κB, indicating that both can act as parallel ‘master transcription factors’ in the signaling network and be co-activated by the same upstream kinase [44]. In addition, some studies have reported signaling axes independent of the classical ROS-NF-κB pathway—for example, nanoparticles can induce macrophage M1 polarization through the ROS-TRAF6-IRF5 pathway, suggesting an NF-κB-independent mechanism for IRF5 activation [45]. IRF5 and NF-κB also cooperate at the transcriptional level—IRF5 can be indirectly recruited to multiple inflammatory gene loci through interaction with the RelA subunit, and downstream of TLR8, their regulation of cytokines involves both cooperation and functional differentiation [46]. Taken together, we speculate that IRF5 may not act merely as a downstream effector of ROS/NF-κB, but rather forms a parallel, cooperative, and functionally complementary regulatory relationship with them within the inflammatory signaling network, which will be the focus of our subsequent mechanistic investigations.

Compared with previous studies that primarily focused on surface modification or single mechanisms, the innovation of this study lies in its systematic approach: for the first time, immunomodulatory activities of Fe_3_O_4_ particles across different particle size ranges were systematically compared under unified preparation and surface coating conditions.

Comprehensive research results indicate that nanoparticle size is a critical parameter in the design of immunomodulatory biomaterials. A size range of approximately 10 nm was identified as the optimal range, providing a rational design principle for iron-based nanotherapeutics and laying a solid foundation for developing combined cancer therapies.

Current research has shown that Fe_3_O_4_ nanocarriers mediate ferroptosis to promote tumor cell apoptosis [21,47]. In this study, we found that iron oxide nanoparticles of different sizes could synergistically promote macrophage M1 polarization to varying degrees, with size-dependent effects observed. However, the active polarization of macrophages driven by Fe_3_O_4_ nanoparticles may be a complex regulatory process, which requires further investigation in our subsequent studies. Literature reports indicate that ferroptosis is involved in regulating macrophage polarization [48,49], which leads us to consider whether ferroptosis plays an important role in the Fe_3_O_4_-driven M1 polarization process. We will explore this in detail in future studies to lay a foundation for the application of Fe_3_O_4_ nanoparticles in tumor therapy.

Meanwhile, based on the key premise confirmed by this study—no direct cytotoxicity at therapeutic concentrations—future research could explore combined strategies involving ferroptosis inducers (Erastin/RSL3) and photothermal therapy (PTT). This synergistic approach is expected to enhance tumor cell lipid peroxidation levels through the local thermal effects generated by PTT, while promoting cellular uptake of ferroptosis inducers and accumulation of reactive oxygen species, thereby achieving a dual enhancement effect of “thermal therapy-chemical sensitization.” Future studies will focus on functional modification of this size-optimized platform to enable targeted delivery and evaluating its synergistic effects with immune checkpoint inhibitors to accelerate clinical translation.

In recent years, research on immune modulation by various nanoparticles has advanced rapidly. Metallic nanoparticles (such as gold and silver) can alleviate inflammation by modulating the NF-κB pathway [50]; lipid nanoparticles (LNPs) serve not only as core delivery vehicles for mRNA vaccines but also exhibit immunoadjuvant activity due to their intrinsic components [51,52]. Polymer nanoparticles (e.g., PLGA) enhance antigen presentation and T-cell activation in tumor immunotherapy owing to their controllable release properties [53]. Biomimetic nanoplatforms, particularly macrophage membrane-coated particles, achieve lesion targeting and immune modulation by leveraging innate immune characteristics [54]. Extracellular vesicles, as low-immunogenicity carriers, demonstrate potential in mRNA delivery and autoimmune disease treatment. These studies provide novel strategies for precise immune modulation of nanoparticles in cancer and inflammatory diseases. However, most of these nanomaterials employ passive delivery systems, limiting spatiotemporal precision control. In contrast, iron oxide (Fe_3_O_4_) nanoparticles enable active targeted delivery and directional recruitment of immune cells under magnetic field guidance [55]. Additionally, Fe_3_O_4_ nanoparticles exhibit unique comprehensive advantages in multifunctional immune modulation and integrated diagnosis/treatment. On one hand, their abundant surface modification sites allow conjugation of multiple immunomodulators for synergistic therapy; their intrinsic enzyme-like activity directly regulates reactive oxygen species levels and immune signaling pathways [56]. On the other hand, Fe_3_O_4_ can synergistically activate systemic antitumor immunity through physical energy conversion methods such as magnetothermal therapy and sonodynamic therapy [57]. More importantly, Fe_3_O_4_ nanoparticles can directly reprogram T cells under magnetic field manipulation, generating CAR-T-like cells in vivo without relying on gene editing, thereby opening new avenues for solid tumor immunotherapy [58]. This multidimensional functional integration endows Fe_3_O_4_ magnetic nanoparticles with remarkable unique potential in precision immune regulation and clinical translation.

This study, of course, has limitations. First, experiments were primarily based on the RAW264.7 cell line. The heterogeneity of human macrophages and the more complex human TME may introduce differences, necessitating validation in primary cells and humanized mouse models in the future. Second, the long-term metabolic fate, biodistribution, and potential toxicity of ~10 nm particles require more detailed pharmacokinetic and toxicological evaluation. Finally, for clinical translation, future work should explore more precise targeted delivery strategies (e.g., functionalization with M2 macrophage or tumor-specific antibodies) and evaluate their combination potential with emerging immunotherapies such as immune checkpoint inhibitors.

## 4. Materials and Methods

### 4.1. Synthesis and Surface Modification of Fe_3_O_4_ Nanoparticles with Controlled Sizes

Fe_3_O_4_ magnetic nanoparticles (MNPs) of four distinct sizes—5 nm, 10 nm, 30 nm, and 100 nm—were synthesized using the polyol method, followed by surface coating with sodium tripolyphosphate (STPP) via electrostatic adsorption to ensure colloidal stability in aqueous media.

Ultrasmall Fe_3_O_4_ Nanoparticles (5 nm-Fe_3_O_4_):

A ferrihydrite precursor was prepared by dissolving sodium hydroxide (NaOH) and ferric chloride hexahydrate (FeCl_3_·6H_2_O) separately in 25 mL of deionized water at a molar ratio of 3:1. The NaOH solution was slowly added to the FeCl_3_ solution under vigorous stirring. The mixture was aged for 30 min at room temperature to form a brown flocculent precipitate. The precipitate was washed three times with ultrapure water and once with ethanol, then dispersed in 50 mL of ethylene glycol. The suspension was transferred to a three-necked flask equipped with a magnetic stirrer and heating condenser, heated at a rate of 2 °C/min to 280 °C ± 2 °C, and maintained at this temperature for 8 h. After cooling to room temperature, a black, homogeneous colloidal suspension of 5 nm Fe_3_O_4_ nanoparticles was obtained.

10 nm Fe_3_O_4_ Nanoparticles:

Iron(III) acetylacetonate (2 mmol) was dissolved in 25 mL of triethylene glycol under magnetic stirring. The solution was transferred to a three-necked flask and heated to 180 °C at a rate of 2 °C/min. After maintaining the temperature between 180 and 200 °C for 10–30 min, the system was rapidly heated to 280 °C and refluxed for 30 min. The resulting suspension was cooled to room temperature, yielding a stable black colloidal solution of 10 nm Fe_3_O_4_ nanoparticles.

30 nm Fe_3_O_4_ Nanoparticles:

Sodium hydroxide and anhydrous ferric chloride (FeCl_3_), at a 3:1 molar ratio, were dissolved in 3.6 mL of deionized water and then mixed with 100 mL of ethylene glycol. The mixture was transferred to a 250 mL three-necked flask and heated to reflux (≈197 °C) at a rate of 2 °C/min. The solution was maintained under reflux for 8 h, then cooled to room temperature to obtain a homogeneous black suspension containing 30 nm Fe_3_O_4_ nanoparticles.

100 nm Fe_3_O_4_ Nanoparticles:

Anhydrous sodium acetate (4.1127 g) and ferric chloride hexahydrate (1.6242 g) were dissolved in 6.48 mL of deionized water. The solution was supplemented with 100 mL of ethylene glycol and homogenized by sonication and stirring for 3 min in a 100 mL beaker. It was then transferred to a three-necked flask and heated to 278 °C at 2 °C/min, followed by 8 h of reflux. After cooling, a black colloidal suspension of 100 nm Fe_3_O_4_ nanoparticles was obtained.

STPP Coating Procedure:

Nanoparticles synthesized via the polyol method are stable in organic solvents (e.g., ethanol) but tend to aggregate upon dilution in aqueous media due to desorption of polyol ligands. To enhance aqueous dispersibility, all particles were coated with biocompatible STPP via electrostatic interaction.

For 30 nm and 100 nm NPs (centrifugation-based purification):

A known volume of colloidal suspension was mixed with an equal volume of anhydrous ethanol and ultrasonicated for 2–5 min to disperse aggregates. After 5 min of settling, the mixture was centrifuged at 8000 rpm for 10 min. The supernatant was discarded, and the precipitate was washed twice with ethanol to remove residual polyols. The purified MNPs were then suspended in deionized water, ultrasonicated for 2–5 min, and centrifuged again under the same conditions to remove residual ethanol. This water-washing step was repeated twice.

STPP (optimized concentration) was dissolved in deionized water via ultrasonication (2–5 min), followed by 5 min of incubation. The STPP solution was added dropwise to the washed nanoparticle suspension under continuous magnetic stirring (300 rpm) for 2 h to ensure complete surface adsorption. Unbound STPP was removed by centrifugation (8000 rpm, 10 min). The final STPP-coated MNPs were redispersed in deionized water and designated as **30 nm-Fe_3_O_4_-STPP** and **100 nm-Fe_3_O_4_-STPP**.

For 5 nm and 10 nm NPs (dialysis-based purification):

Due to their small size and low sedimentation coefficients, centrifugation and magnetic separation were ineffective. Thus, dialysis was employed.

2 mL of the colloidal suspension (us-Fe_3_O_4_ or 10 nm-Fe_3_O_4_) was mixed with 8 mL of an aqueous STPP solution (55 mg in deionized water) under magnetic stirring (300 rpm) for 2 h. The mixture was then sealed in a dialysis bag (molecular weight cutoff: 14,000 Da) and dialyzed against deionized water for 48 h. The dialysate was replaced every 4 h during the first 24 h, then every 6 h thereafter to ensure efficient removal of excess STPP and residual polyols. The retained colloidal solution was collected and labeled as us-Fe_3_O_4_-STPP and 10 nm-Fe_3_O_4_-STPP.

### 4.2. Physicochemical Characterization of Fe_3_O_4_ Nanoparticles

The physicochemical properties of the synthesized nanoparticles were characterized using the following techniques:

X-ray diffraction (XRD): Patterns were recorded on a Rigaku D/max-γB diffractometer using CuKα radiation (λ = 0.154056 nm).

Transmission electron microscopy (TEM): Images and selected-area electron diffraction (SAED) patterns were obtained using the JEM-2100F Transmission Electron Microscope (200 kV) manufactured by JEOL Ltd., (Tokyo, Japan). Samples were prepared by dropping diluted nanoparticle suspensions onto carbon-coated copper grids and allowing them to air-dry.

Fourier-transform infrared spectroscopy (FT-IR): Spectra were acquired using a PerkinElmer Spectrum One spectrometer (Thermo Nicolet Corporation Nicolet 510P Fourier-transform infrared spectrometer manufactured by Thermo Nicolet Corporation, Madison, WI, USA) in the range of 400–4000 cm^−1^.

Hydrodynamic size and zeta potential: Measured using a Malvern Zetasizer Nano S90 (Malvern Instruments Zetasizer Nano ZS Laser Dynamic Light Scatter (He/Ne laser, λ = 633 nm) manufactured by Malvern Instruments, Malvern, UK) after dispersing STPP-coated nanoparticles in deionized water. All measurements were performed in triplicate.

### 4.3. Cell Culture

Human immortalized keratinocytes (HaCaT) and murine monocyte/macrophage leukemia cells (RAW264.7) were cultured at 37 °C in a humidified atmosphere with 5% CO_2_.

HaCaT cells were maintained in Dulbecco’s Modified Eagle Medium (DMEM), high glucose, supplemented with 10% fetal bovine serum (FBS) and 1% antibiotic-antimycotic solution.

RAW264.7 cells were cultured in Roswell Park Memorial Institute (RPMI)-1640 medium supplemented with 10% FBS.

All cell lines were passaged every 2–3 days and used during exponential growth phase.

### 4.4. Cytotoxicity Assay (MTT Assay)

The cytotoxicity of STPP-coated Fe_3_O_4_ nanoparticles toward HaCaT cells was evaluated using the MTT [(3-(4,5-dimethylthiazol-2-yl)-2,5-diphenyltetrazolium bromide)] assay. Cells in logarithmic phase were seeded into 96-well plates at a density of 8 × 10^3^ cells per well. After 24 h of adhesion, the culture medium was replaced with fresh medium containing Fe_3_O_4_-STPP nanoparticles at concentrations of 6.25, 12.5, 25, and 50 µg/mL. Cells were incubated for an additional 24 h, followed by addition of 10 µL of MTT solution (5 mg/mL, filter-sterilized through 0.22 µm membrane). After 4 h of incubation, the supernatant was carefully removed, and 150 µL of dimethyl sulfoxide (DMSO) was added to dissolve the formed formazan crystals. The absorbance was measured at 490 nm using a microplate reader. Cell viability (%) was calculated relative to untreated control cells.

### 4.5. Cellular Uptake of Fe_3_O_4_ Nanoparticles

Quantitative Analysis (ICP-OES, Shimadzu ICPE9820, Kyoto, Japan):

RAW264.7 cells were seeded in 6-well plates and treated with Fe3O4-STPP nanoparticles (6.25 µg/mL) for 0.5 h or 2 h. After treatment, cells were washed five times with phosphate-buffered saline (PBS) to remove uninternalized nanoparticles. Cells were then lysed in 1 mL of deionized water by adding 100 µL of 1% (*v*/*v*) nitric acid and sonication (30 s, 4 °C). The lysates were diluted 1:1 with deionized water, filtered through 0.22 µm membranes, and analyzed for iron content using inductively coupled plasma optical emission spectrometry (ICP-OES).

Fluorescence Imaging (Zeiss LSM 710 Laser Confocal Microscope, Jena, Germany):

To visualize nanoparticle uptake, STPP-coated Fe_3_O_4_ nanoparticles were modified with polyethyleneimine (PEI) via electrostatic interaction to form PEI-PPS-NPs. Cy5.5 dye (1 mg/mL in anhydrous DMSO) was conjugated to PEI-modified nanoparticles under vortexing at pH 7.4 and incubated at 37 °C for 2 h on a shaker. Fluorescently labeled nanoparticles (designated as **Cy5.5-Fe_3_O_4_-STPP**) were collected using a strong magnetic stand, washed three times with PBS, and resuspended in ultrapure water. RAW264.7 cells were incubated with 10 µg/mL Cy5.5-Fe_3_O_4_-STPP for 2 h, washed thoroughly with PBS, and imaged using a confocal laser scanning microscope (excitation: 649 nm; emission: 670 nm).

### 4.6. Functional Assays for Macrophage Polarization

#### 4.6.1. Nitric Oxide (NO) Detection

NO production was measured using a Griess Reagent Kit. RAW264.7 cells were seeded in 96-well plates at 8 × 104 cells/mL. After 24 h, cells were treated with STPP-coated nanoparticles (6.25 µg/mL) and incubated for 8, 16, or 24 h. Culture supernatants (50 µL) were mixed with an equal volume of Griess reagent. Absorbance at 540 nm was measured using a microplate reader (TECAN, Spark Cyto, Männedorf, Switzerland), and NO concentration was calculated against a sodium nitrite standard curve.

#### 4.6.2. Flow Cytometry for CD86 Expression

RAW264.7 cells were seeded in 96-well plates at 7 × 103 cells/well. After 24 h, cells were treated with 6.25 µg/mL of different-sized nanoparticles for 24 h. Cells were harvested, washed with PBS, and stained with fluorescein isothiocyanate (FITC)-conjugated anti-CD86 antibody (1:200 dilution) for 30 min at 4 °C in the dark. After washing, cells were resuspended in PBS and analyzed by flow cytometry (BD FACSCANTO II, BD Biosciences, San Jose, CA, USA), with data processed using FlowJo software(V10). CD86+ cell percentage was determined relative to isotype control.

#### 4.6.3. Scanning Electron Microscopy (SEM)

RAW264.7 cells treated with nanoparticles (6.25 µg/mL, 24 h) were fixed in 2.5% glutaraldehyde (4 °C, 4 h), dehydrated through a graded ethanol series (30–100%), and processed via critical point drying. Samples were sputter-coated with gold and imaged using a scanning electron microscope.

#### 4.6.4. Enzyme-Linked Immunosorbent Assay (ELISA)

Levels of tumor necrosis factor-alpha (TNF-α) and interleukin-6 (IL-6) in culture supernatants were quantified after 24 h of nanoparticle treatment using ELISA kits (Mouse Tumor Necrosis Factor-α (TNF-α) ELISA Research Kit and Mouse Interleukin-6 (IL-6) ELISA Research Kit), following the manufacturer’s instructions. Absorbance was read at 450 nm using a microplate reader.

#### 4.6.5. Intracellular ROS Detection

RAW264.7 cells were seeded in 6-well plates at 1.5 × 10^5^ cells/mL. After 24 h, cells were treated with nanoparticles or the positive control ROSUP (150 µM H_2_O_2_) for 24 h. Cells were then incubated with 10 µM 2′,7′-dichlorodihydrofluorescein diacetate (DCFH-DA) at 37 °C for 20 min in the dark. After washing with PBS, fluorescent images were captured using a fluorescence microscope (excitation: 488 nm; emission: 525 nm).

#### 4.6.6. Quantitative Real-Time PCR (qRT-PCR)

Total RNA was extracted using SPARKeasy Tissue/Cell RNA Rapid Extraction Kit (with genomic DNA removal column) (Sparkjade, Jinan, China), reverse-transcribed into cDNA using a SPARKscript II RT Plus Kit (With gDNA Eraser) (Sparkjade) and amplified using 2xUniversal SYBR Green qPCR Mix (ABclonal, Woburn, MA, USA) on a QuantStudio 6 Flex real-time PCR system (Thermo Fisher Scientific, Waltham, MA, USA). The primers sequence of CD206 and β-actin were list in Table 1. Gene expression levels of CD206 and β-actin (internal control) were quantified. Relative mRNA expression was calculated using the 2^−ΔΔCt^ method.

#### 4.6.7. Western Blotting

Cells were lysed in RIPA buffer containing protease and phosphatase inhibitors. Protein concentrations were determined using a BCA standard solution (5 mg/mL, prepared using bovine serum albumin, Shanghai Yuanye Bio-Technology Co., Ltd., Shanghai, China). Equal amounts of protein (20–30 µg) were separated by 10% SDS-PAGE and transferred onto PVDF membranes. Membranes were blocked in 5% non-fat milk for 1 h, then incubated with primary antibodies against CD86, TLR4, NF-κB p65, MyD88, and IκBα (1:1000 dilution, overnight at 4 °C). After washing, HRP-conjugated secondary antibodies were applied (1:5000, 1 h, RT). Protein bands were visualized using enhanced chemiluminescence (ECL) reagent and imaged with a ChemiDoc Imaging System (Bio-Rad Laboratories, Hercules, CA, USA). Band intensity was quantified using Image J-win 64 v1.54 software.

### 4.7. Preparation of Conditioned Mediums (CMs)

Macrophages were treated with Fe_3_O_4_ nanoparticles of different particle sizes for 24 h. Subsequently, the treated macrophages were cultured in serum-free medium for an additional 24 h. After incubation, the cell culture supernatant was collected by centrifugation and designated as the conditioned medium for subsequent experiments.

### 4.8. Statistical Analysis

All data are presented as mean ± standard deviation (SD). Statistical analyses were performed using GraphPad Prism 6.02. One-way analysis of variance (ANOVA) followed by Tukey’s multiple comparisons test was used to assess differences between multiple groups. A ‘*p*’-value < 0.05 was considered statistically significant.

## 5. Conclusions

In summary, this study successfully engineered size-controlled Fe_3_O_4_ nanoparticles through a reproducible polyol synthesis and stabilized them in aqueous environments via STPP coating. We systematically demonstrated that nanoparticle size critically dictates macrophage polarization outcomes, with 10 nm Fe_3_O_4_-STPP nanoparticles exhibiting the most potent ability to reprogram TAMs toward an antitumor M1 phenotype. This size-optimal effect arises from a well-defined cascade: enhanced cellular uptake → lysosomal iron release → Fenton reaction-driven ROS burst → activation of the TLR4/MyD88/NF-κB signaling axis → transcriptional upregulation of pro-inflammatory cytokines and surface markers (e.g., CD86, TNF-α, IL-6).

This study established a quantitative structure-activity relationship for Fe_3_O_4_ nanomaterials in immunomodulation and proposed a rational design principle: approximately 10 nm was identified as the optimal size for maximizing intracellular iron delivery, reactive oxygen species signaling, and immune reprogramming. This discovery not only provides a novel paradigm for the design of iron-based nanotherapeutic agents but also lays the foundation for developing next-generation combination immunotherapies targeting drug-resistant solid tumors.

## Figures and Tables

**Table 1:** Primers sequence of cytokines.

Cytokines	Forward Sequence (5′-3′)	Reverse Sequence (5′-3′)
CD206	CTCTGTTCAGCTATTGGACGC	CGGAATTTCTGGGATTCAGCTTC
β-actin	AGCCTTCCTTCTTGGGTATGGA	ACGGATGTCAACGTCACACTTA

## Data Availability

Data will be made available on request.

## References

[1] Biswas S.K., Mantovani A. (2010). Macrophage plasticity and interaction with lymphocyte subsets: Cancer as a paradigm. Nat. Immunol..

[2] Nascimento C.S., Alves E.A.R., de Melo C.P., Corrêa-Oliveira R., Calzavara-Silva C.E. (2021). Immunotherapy for cancer effects of iron oxide nanoparticles on polarization of tumor-associated macrophages. Nanomedicine.

[3] Rao L., Wu L., Liu Z., Tian R., Yu G., Zhou Z., Yang K., Xiong H.G., Zhang A., Yu G.T. (2020). Hybrid cellular membrane nanovesicles amplify macrophage immune responses against cancer recurrence and metastasis. Nat. Commun..

[4] Wu J., Lu H., Xu X., Rao L., Ge Y. (2024). Engineered Cellular Vesicles Displaying Glycosylated Nanobodies for Cancer Immunotherapy. Angew. Chem. Int. Ed..

[5] Khan I., Saeed K., Khan I. (2019). Nanoparticles: Properties, applications and toxicities. Arab. J. Chem..

[6] Dolai J., Mandal K., Jana N.R. (2021). Nanoparticle Size Effects in Biomedical Applications. ACS Appl. Nano Mater..

[7] Komatsuzaki A., Ohyanagi T., Tsukasaki Y., Miyanaga Y., Ueda M., Jin T. (2015). Compact halo-ligand-conjugated quantum dots for multicolored single-molecule imaging of overcrowding GPCR proteins on cell membranes. Small.

[8] Sapsford K.E., Algar W.R., Berti L., Gemmill K.B., Casey B.J., Oh E., Stewart M.H., Medintz I.L. (2013). Functionalizing nanoparticles with biological molecules: Developing chemistries that facilitate nanotechnology. Chem. Rev..

[9] Elsabahy M., Wooley K.L. (2012). Design of polymeric nanoparticles for biomedical delivery applications. Chem. Soc. Rev..

[10] Lu A.H., Salabas E.L., Schuth F. (2007). Magnetic nanoparticles: Synthesis, protection, functionalization, and application. Angew. Chem. Int. Ed..

[11] Su Y., Wang Z., Hu Q., Sun Y., Dong Q., Piao Y., Hua Z., Dong C., Hu H., Shen Y. (2026). Screening of transcytosable iron oxide nanoparticles (TIONs) for deep tissue-penetrating imaging. Biomaterials.

[12] Liu L., Wang Y., Guo X., Zhao J., Zhou S. (2020). A Biomimetic Polymer Magnetic Nanocarrier Polarizing Tumor-Associated Macrophages for Potentiating Immunotherapy. Small.

[13] Lyu B., Chen J., Jiang H., Cui B., Liu X., Zhang X., Long X., Chen Z., Sun Y., Ge D. (2026). Polynorepinephrine nanoagent enables targeted mitochondrial delivery for enhanced tumor therapy through ferroptosis. Colloids Surf. B Biointerfaces.

[14] Fato T.P., Dit Adama N’goran K.P., Kinimo K.C., Aliou Guillaume P.L., Fodjo E.K., Diabate D. (2026). Recent advances in magnetite (Fe_3_O_4_) nanoparticles: Sustainable synthesis, surface engineering, and emerging environmental applications. Environ. Nanotechnol. Monit. Manag..

[15] Álvarez-Gil L., Soto-Calle G., Lopera A., Becerra A., Navarro-Gallón S., Rojas-Reyes N.R. (2025). Evaluation of conditions favorable for the enhanced stability of magnetite suspensions using visible spectroscopy. Colloid Polym. Sci..

[16] Araujo E.V., Carneiro S.V., Neto D.M.A., Freire T.M., Costa V.M., Freire R.M., Fechine L.M.U.D., Clemente C.S., Denardin J.C., dos Santos J.C.S. (2024). Advances in surface design and biomedical applications of magnetic nanoparticles. Adv. Colloid Interface Sci..

[17] Murari K., Vijayakumar Y. (2026). CTAB-Assisted Synthesis of Iron Oxide Nanoparticles for Enhanced Photocatalytic Dye Degradation. Phys. Status Solidi A-Appl. Mater. Sci..

[18] Li X., Li W., Wang M., Liao Z. (2021). Magnetic nanoparticles for cancer theranostics: Advances and prospects. J. Control. Release.

[19] Serga V., Burve R., Maiorov M., Krumina A., Skaudzius R., Zarkov A., Kareiva A., Popov A. (2020). Impact of Gadolinium on the Structure and Magnetic Properties of Nanocrystalline Powders of Iron Oxides Produced by the Extraction-Pyrolytic Method. Materials.

[20] Riaz S., Ali S., Summer M., Akhtar U., Noor S., Haqqi R., Farooq M.A., Sardar I. (2025). Multifunctional Magnetic Nanoparticles for Targeted Drug Delivery Against Cancer: A Review of Mechanisms, Applications, Consequences, Limitations, and Tailoring Strategies. Ann. Biomed. Eng..

[21] Li Y., Chen J., Xia Q., Shang J., He Y., Li Z., Chen Y., Gao F., Yu X., Yuan Z. (2024). Photothermal Fe_3_O_4_ nanoparticles induced immunogenic ferroptosis for synergistic colorectal cancer therapy. J. Nanobiotechnol..

[22] Zanganeh S., Hutter G., Spitler R., Lenkov O., Mahmoudi M., Shaw A., Pajarinen J.S., Nejadnik H., Goodman S., Moseley M. (2016). Iron oxide nanoparticles inhibit tumour growth by inducing pro-inflammatory macrophage polarization in tumour tissues. Nat. Nanotechnol..

[23] Li K., Lu L., Xue C., Liu J., He Y., Zhou J., Xia Z., Dai L., Luo Z., Mao Y. (2020). Polarization of tumor-associated macrophage phenotype via porous hollow iron nanoparticles for tumor immunotherapy in vivo. Nanoscale.

[24] Gu Z., Liu T., Tang J., Yang Y., Song H., Tuong Z.K., Fu J., Yu C. (2019). Mechanism of Iron Oxide-Induced Macrophage Activation: The Impact of Composition and the Underlying Signaling Pathway. J. Am. Chem. Soc..

[25] Zhao H., Zhao B., Wu L., Xiao H., Ding K., Zheng C., Song Q., Sun L., Wang L., Zhang Z. (2019). Amplified Cancer Immunotherapy of a Surface-Engineered Antigenic Microparticle Vaccine by Synergistically Modulating Tumor Microenvironment. ACS Nano.

[26] Li C.X., Zhang Y., Dong X., Zhang L., Liu M.D., Li B., Zhang M.K., Feng J., Zhang X.Z. (2019). Artificially Reprogrammed Macrophages as Tumor-Tropic Immunosuppression-Resistant Biologics to Realize Therapeutics Production and Immune Activation. Adv. Mater..

[27] Di Santo M., D’Antoni C., Rubio A., Alaimo A., Pérez O. (2021). Chitosan-tripolyphosphate nanoparticles designed to encapsulate polyphenolic compounds for biomedical and pharmaceutical applications—A review. Biomed. Pharmacother..

[28] Le N., Steinbring C., Le-Vinh B., Jalil A., Matuszczak B., Bernkop-Schnürch A. (2021). Polyphosphate coatings: A promising strategy to overcome the polycation dilemma. J. Colloid Interface Sci..

[29] Bekic M., Tomic S., Rudolf R., Milanovic M., Vucevic D., Anzel I., Colic M. (2019). The Effect of Stabilisation Agents on the Immunomodulatory Properties of Gold Nanoparticles Obtained by Ultrasonic Spray Pyrolysis. Materials.

[30] Wan J., Yuan R., Zhang C., Wu N., Yan F., Yu S., Chen K. (2016). Stable and Biocompatible Colloidal Dispersions of Superparamagnetic Iron Oxide Nanoparticles with Minimum Aggregation for Biomedical Applications. J. Phys. Chem. C.

[31] Chen X., Zheng X., Yu X., Li X., Lin Y., Lin H., Ye S., Huang X., Tang D., Lai W. (2023). Novel rapid coordination of ascorbic acid 2-phosphate and iron(III) as chromogenic substrate system based on Fe_2_O_3_ nanoparticle and application in immunoassay for the colorimetric detection of carcinoembryonic antigen. Talanta.

[32] Velazquez-Albino A.C., Elsea B., Melnyk A., Eswaran N., Imhoff E.D., Williams A.G., Graham W., Johnson J.A., Johnson C.E., Butala M.M. (2026). Esterification synthesis of iron oxide nanoparticle tracers for magnetic particle imaging (MPI). Nanoscale.

[33] Mano S., Kanehira K., Sonezaki S., Taniguchi A. (2014). Toll-Like Receptor 4 Is Involved in Titanium Dioxide Nanoparticle Incorporation into Cells. Sci. Adv. Mater..

[34] Hua Y., Wu J., Wu H., Su C., Li X., Ao Q., Zeng Q., Zhu X., Zhang X. (2021). Exposure to hydroxyapatite nanoparticles enhances Toll-like receptor 4 signal transduction and overcomes endotoxin tolerance in vitro and in vivo. Acta Biomater..

[35] Qian X., Zhang J., Gu Z., Chen Y. (2019). Nanocatalysts-augmented Fenton chemical reaction for nanocatalytic tumor therapy. Biomaterials.

[36] Wang B., Zhang X., Wang Z., Shi D. (2020). Ferroptotic nanomaterials enhance cancer therapy via boosting Fenton-reaction. J. Drug Deliv. Sci. Technol..

[37] Sudduth E., Ruiz A., Trautmann-Rodriguez M., Fromen C. (2024). Age-dependent changes in phagocytic activity: In vivo response of mouse pulmonary antigen presenting cells to direct lung delivery of charged PEGDA nanoparticles. J. Nanobiotechnol..

[38] Liao Z., Wen Z., Chen J., Gu J., Su H., Zhang X., Huo S. (2025). Size-dependent ultrasound enhancement of nanoparticle endocytosis in tumor cells. Ultrason. Sonochem..

[39] Claudia M., Kristin Ö., Jennifer O., Eva R., Eleonore F. (2017). Comparison of fluorescence-based methods to determine nanoparticle uptake by phagocytes and non-phagocytic cells in vitro. Toxicology.

[40] Deng Z., Wan J., Shan S., Wang J., Geng L., Yue Y., Zhang Q., Wu N. (2025). Delivery of 5-fluorouracil modified MicroRNAs by fucoidan-based magnetic nanoparticles to enhances tumor-localized immunotherapy and chemotherapy. Mater. Today Adv..

[41] Sun M., Gu P., Yang Y., Yu L., Jiang Z., Li J., Le Y., Chen Y., Ba Q., Wang H. (2021). Mesoporous silica nanoparticles inflame tumors to overcome anti-PD-1 resistance through TLR4-NFκB axis. J. Immunother. Cancer.

[42] Mosser D.M., Edwards J.P. (2008). Exploring the full spectrum of macrophage activation. Nat. Rev. Immunol..

[43] Hedl M., Yan J., Witt H., Abraham C. (2019). IRF5 Is Required for Bacterial Clearance in Human M1-Polarized Macrophages, and IRF5 Immune-Mediated Disease Risk Variants Modulate This Outcome. J. Immunol..

[44] Lopez-Pelaez M., Lamont D.J., Peggie M., Shpiro N., Gray N.S., Cohen P. (2014). Protein kinase IKKβ-catalyzed phosphorylation of IRF5 at Ser462 induces its dimerization and nuclear translocation in myeloid cells. Proc. Natl. Acad. Sci. USA.

[45] Zheng Y., Han Y., Wang T., Liu H., Sun Q., Hu S., Chen J., Li Z. (2022). Reprogramming Tumor-Associated Macrophages via ROS-Mediated Novel Mechanism of Ultra-Small Cu_2−x_Se Nanoparticles to Enhance Anti-Tumor Immunity. Adv. Funct. Mater..

[46] Stein T., Wollschlegel A., Te H., Weiss J., Joshi K., Kinzel B., Billich A., Guntermann C., Lehmann J. (2018). Interferon regulatory factor 5 and nuclear factor kappa-B exhibit cooperating but also divergent roles in the regulation of pro-inflammatory cytokines important for the development of TH1 and TH17 responses. FEBS J..

[47] Szwed M., Poczta-Krawczyk A., Bukowski K., Marczak A. (2025). Nanoparticle-Mediated Ferroptosis for Cancer Therapy: Mechanisms and Therapeutic Strategies. Nanotechnol. Sci. Appl..

[48] Gao J., Zhang X., Liu Y., Gu X. (2025). Ferroptosis in immune cells: Implications for tumor immunity and cancer therapy. Cytokine Growth Factor Rev..

[49] Hu J., Cui L., Hou B., Ding X., Liu H., Sun W., Mi Y., Chen Y., Zou Z. (2025). Ferroptosis in tumor associated immune cells: A double-edged sword against tumors. Crit. Rev. Oncol. Hematol..

[50] Barik P., Mondal S. (2025). Immunomodulatory effects of metal nanoparticles: Current trends and future prospects. Nanoscale.

[51] Szebeni J. (2025). Unique Features and Collateral Immune Effects of mRNA-LNP COVID-19 Vaccines: Plausible Mechanisms of Adverse Events and Complications. Pharmaceutics.

[52] Gu J., Xu Z., Liu Q., Tang S., Zhang W., Xie S., Chen X., Chen J., Yong K., Yang C. (2024). Building a Better Silver Bullet: Current Status and Perspectives of Non-Viral Vectors for mRNA Vaccines. Adv. Healthc. Mater..

[53] Wang Y., Huang P., Li C., Tu S., Yang H. (2025). PLGA-based nanoparticles in colorectal cancer immunotherapy: Current concepts and future perspectives. Front. Pharmacol..

[54] Hou Y., Wu R., Zhou Y., Yin C., Gai Y., Jiang D., Wang K., Xia X. (2025). Macrophage membrane-coated nanoparticles in inflammatory diseases: From bioinspired design to translational potential. J. Nanobiotechnol..

[55] Yang C., Li S., Wang L. (2025). Engineered iron oxide nanoplatforms: Reprogramming immunosuppressive niches for precision cancer theranostics. Mol. Cancer.

[56] Huang Y., Cai X., Li Y., Zhang M., Sheng J., Gu N. (2025). The metabolic fate of iron-based magnetic nanomaterials and their impact on macrophage function. Magn. Med..

[57] Liu H., Yang X., Liang X., Xia Y. (2025). Dual-magnetically driven nanozymes for glioblastoma immunotherapy via magnetothermal and NIR-amplified ferroptosis and apoptosis. Mater. Today Bio.

[58] Qiu Y., Wu H., Lin Z., Chen J., Tian S., Wang Z., Ran H., Wang Y., Cheng L. (2025). Magneto-photo-acoustic Nanotheranostics Orchestrate Ferroptosis-Immune Cross Talk for Spatiotemporally Amplified Triple-Negative Breast Cancer Therapy. Biomater. Res..

